# Kisspeptin Administration Stimulates Reproductive Hormones but Does Not Affect Anxiety in Humans

**DOI:** 10.1210/clinem/dgaf128

**Published:** 2025-02-26

**Authors:** Edouard G Mills, Layla Thurston, Lisa Yang, Tia Hunjan, Maria Phylactou, Bijal Patel, Sophie A Clarke, Chioma Izzi-Engbeaya, Jovanna Tsoutsouki, Megan Young, Paul Bech, Natalie Ertl, Matthew B Wall, Ali Abbara, Alexander N Comninos, Waljit S Dhillo

**Affiliations:** Section of Endocrinology and Investigative Medicine, Imperial College London, London W12 0NN, UK; Department of Endocrinology, Imperial College Healthcare NHS Trust, London W6 8RF, UK; Section of Endocrinology and Investigative Medicine, Imperial College London, London W12 0NN, UK; Section of Endocrinology and Investigative Medicine, Imperial College London, London W12 0NN, UK; Section of Endocrinology and Investigative Medicine, Imperial College London, London W12 0NN, UK; Section of Endocrinology and Investigative Medicine, Imperial College London, London W12 0NN, UK; Department of Endocrinology, Imperial College Healthcare NHS Trust, London W6 8RF, UK; Section of Endocrinology and Investigative Medicine, Imperial College London, London W12 0NN, UK; Section of Endocrinology and Investigative Medicine, Imperial College London, London W12 0NN, UK; Section of Endocrinology and Investigative Medicine, Imperial College London, London W12 0NN, UK; Department of Endocrinology, Imperial College Healthcare NHS Trust, London W6 8RF, UK; Section of Endocrinology and Investigative Medicine, Imperial College London, London W12 0NN, UK; Section of Endocrinology and Investigative Medicine, Imperial College London, London W12 0NN, UK; Section of Endocrinology and Investigative Medicine, Imperial College London, London W12 0NN, UK; Section of Endocrinology and Investigative Medicine, Imperial College London, London W12 0NN, UK; Clinical Imaging Services, Perceptive London, London W12 0NN, UK; Section of Endocrinology and Investigative Medicine, Imperial College London, London W12 0NN, UK; Clinical Imaging Services, Perceptive London, London W12 0NN, UK; Section of Endocrinology and Investigative Medicine, Imperial College London, London W12 0NN, UK; Department of Endocrinology, Imperial College Healthcare NHS Trust, London W6 8RF, UK; Section of Endocrinology and Investigative Medicine, Imperial College London, London W12 0NN, UK; Department of Endocrinology, Imperial College Healthcare NHS Trust, London W6 8RF, UK; Section of Endocrinology and Investigative Medicine, Imperial College London, London W12 0NN, UK; Department of Endocrinology, Imperial College Healthcare NHS Trust, London W6 8RF, UK

**Keywords:** kisspeptin, behavior, anxiety, reproduction

## Abstract

**Context:**

Kisspeptin is a critical endogenous activator of the reproductive system, with escalating clinical interest as a novel therapeutic for common reproductive and psychosexual disorders. However, conflicting animal data suggest that kisspeptin can have anxiolytic, neutral, or anxiogenic effects.

**Objective:**

Given the rapid development of kisspeptin-based therapeutics, it is important to comprehensively investigate the effects of kisspeptin administration on behavioral, biochemical, and physiological measures of anxiety in humans.

**Methods:**

Ninety-five participants (N = 63 male, N = 32 female) completed a double-blind, randomized, placebo-controlled, crossover protocol (mean age ± SEM 30.9 ± 0.9 y, body mass index 24.0 ± 0.4), attending both for a 75-minute intravenous kisspeptin-54 infusion (1 nmol/kg/h) and rate-matched placebo (in random order). Behavioral, biochemical, and physiological measures of anxiety were compared between kisspeptin and placebo visits, using a state-anxiety psychometric questionnaire before and at the end of the infusions, and blood sampling (for reproductive hormones and cortisol) and heart rate measurements at 15-minute intervals. Blood pressure assessment took place before and at the end of the infusions.

**Results:**

Kisspeptin administration robustly increased serum luteinizing hormone to similar levels previously described using this administration protocol, confirming that the dose was biologically active (*P* < .001). State anxiety was not significantly altered by kisspeptin, compared to placebo (*P* = .13). Moreover, kisspeptin had no significant effects on circulating cortisol (*P* = .73), systolic (*P* = .74) or diastolic blood pressure (*P* = .90), or heart rate (*P* = .52).

**Conclusion:**

This is the first study demonstrating that a biologically active dose of kisspeptin to men and women does not affect behavioral, biochemical, or physiological measures of anxiety. Given that animal studies have yielded contradictory results, this provides key clinical data and reassurance that kisspeptin does not induce anxiety in humans and so informs the current development of kisspeptin-based therapeutics for common reproductive and psychosexual disorders.

The neuropeptide kisspeptin (encoded by the nonhuman *Kiss1* and human *KISS1* genes) sits at the apex of the reproductive axis, where it plays a crucial role in regulating downstream reproductive hormone secretion (1). Here, kisspeptin acts via the kisspeptin receptor on hypothalamic gonadotropin-releasing hormone (GnRH) neurons to stimulate the release of GnRH (2). This in turn controls downstream gonadal function via luteinizing hormone (LH) and follicle-stimulating hormone. Due to this key role in regulating physiological reproductive hormone secretion, there has been escalating clinical interest in using kisspeptin-based medicines to restore hormonal secretion in common reproductive disorders (3), including hypothalamic amenorrhea (4-7), hyperprolactinemia (8, 9), obesity-related hypogonadism (10), and as a safer trigger for inducing oocyte maturation in fertilization performed in vitro (11-14). Moreover, clinical attention has further increased in response to recent evidence highlighting kisspeptin as a novel strategy for treating low bone mineral density (15, 16) and metabolic dysfunction–associated steatotic liver disease (17), demonstrating widespread therapeutic potential.

Besides the hypothalamus, kisspeptin and its receptor are extensively expressed in numerous corticolimbic brain structures in rodents (18) and humans (19-21), with emerging functional evidence from preclinical animal models (22-24) and human studies (25-29) revealing that kisspeptin-signaling has important roles in modulating reproductive behavior. Translating these findings into patient benefit, we have recently demonstrated that kisspeptin administration to women (30) and men (31) with low sexual desire robustly enhances sexual brain activity with associated improvements in sexual desire and a proerectile effect in men. Therefore, in addition to common reproductive disorders, kisspeptin-based therapeutics also have putative utility in the treatment of psychosexual disorders.

It is notable that unlike the evidence revealing an unambiguous and positive modulatory effect for kisspeptin in reproductive hormone release and behavior, its influence on anxiety remains uncertain (32). Indeed, contradictory preclinical animal data suggest that kisspeptin can have anxiolytic (23, 33), neutral (34, 35), or anxiogenic effects (36-38). Hence, given the rapid development of kisspeptin-based therapeutics for an accelerating number of clinical indications, it is imperative to elucidate kisspeptin's effects on anxiety in humans. Herein, we report the largest clinical study comprehensively investigating the effects of kisspeptin administration on behavioral, biochemical, and physiological measures of anxiety in humans.

## Materials and Methods

### Study Approval

The present study reports a post hoc analysis of 95 male and female participants investigating the clinical effects of kisspeptin administration on anxiety. The participants were recruited to research studies examining the effects of kisspeptin administration on human brain processing, as reported in (25, 30, 31). All participants provided informed written consent before inclusion, with studies performed in accordance with the principles of the Declaration of Helsinki. Ethical approval was gained from the West London (ref: 04/Q0406/151) and Riverside (ref: 17/LO/1504) regional ethics committees.

### Participants

Potential participants were invited to take part through advertisements placed online and in local newspapers, and underwent a detailed medical screening visit, including medical history, medication history, clinical examination, electrocardiogram, and blood tests to confirm normal health status and exclude any endocrine abnormalities. All participants were healthy and were free of any history of medical or additional psychological conditions beyond prespecified conditions (inclusion and exclusion criteria as per (25, 30, 31)). All female participants were premenopausal with regular menstrual cycle lengths between 28 and 35 days and not taking any form of hormonal contraception. Participants were excluded based on the following additional criteria: use of prescription, recreational, or investigational drugs in the preceding 6 months; or blood donation within 3 months of study participation. Male and female participants with acquired and generalized hypoactive sexual desire disorder were diagnosed according to the International Classification of Diseases 11th Revision criteria (39), did not have any endocrine abnormalities, and were otherwise free of current or past psychiatric illness (including clinical anxiety and depression as determined using the Generalized Anxiety Disorder Questionnaire-7 (40) and Patient Health Questionnaire-9 [PHQ-9] (41), respectively, with all scores below threshold for anxiety and depressive disorders).

Following screening and informed consent, 63 male and 32 female participants took part in the study, with all participants completing both study visits.

### Study Design

**Figure 1.**
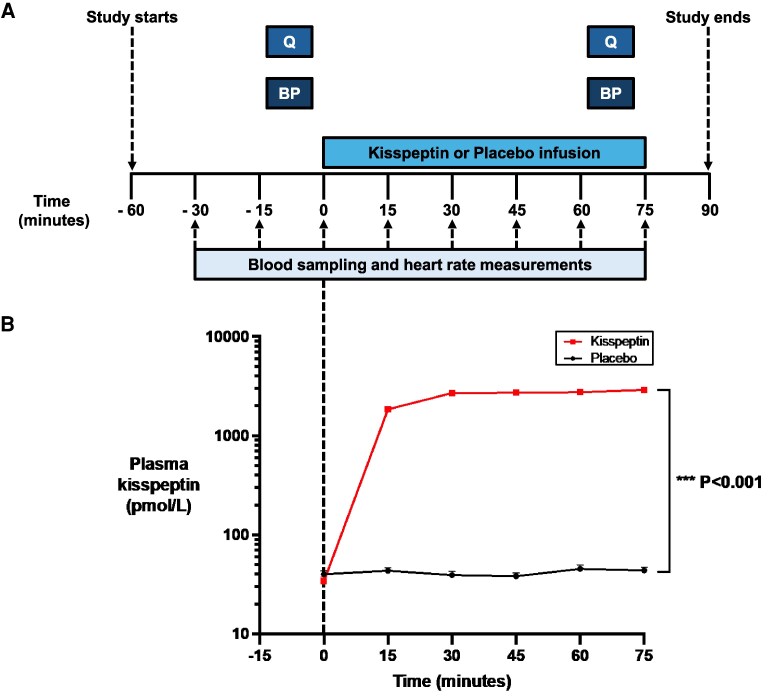
Protocol schematic and effects of kisspeptin administration on circulating kisspeptin levels. A, Ninety-five eugonadal participants completed a randomized, double-blind, 2-way crossover, placebo-controlled protocol. Participants attended for 2 study visits each, in random order: once for a 75-minute intravenous kisspeptin-54 infusion (1 nmol/kg/h) and again for a rate-matched placebo. Participants completed psychometric questionnaires (Q) and had blood pressure measurements (BP) before and toward the end of kisspeptin and placebo administration. Blood sampling (to measure circulating reproductive hormone and cortisol levels) and heart rate measurements took place at 15-minute intervals from −30 to 75 minutes. B, Kisspeptin administration resulted in increased circulating kisspeptin levels reaching a plateau at 30 minutes after initiation. Therefore, circulating kisspeptin levels were stable during the intrainfusion psychometric assessments. Groups were compared by 2-way analysis of variance with Bonferroni multiple comparison test. Data depict mean ± SEM. N = 95 (N = 63 male participants and N = 32 female participants).

Participants completed a randomized, double-blind, 2-way crossover, placebo-controlled protocol (Fig. 1A), with participants attending 2 study visits each (1 for kisspeptin administration and 1 for placebo administration). To ensure washout, study visits were at least 7 days apart for male participants, given that the circulating half-life of kisspeptin-54 is 27.6 minutes (42). Study visits for female participants were conducted in the follicular phase of the menstrual cycle (ie, days 2-7 inclusive) to control for changes in reproductive hormones over the course of the cycle and were conducted during 2 different menstrual cycles (ie, 1 visit per menstrual cycle). The crossover design, in which participants acted as their own control, minimized interparticipant variation and enhanced power. The order of the interventions was randomized using an independent web-based randomization platform (www.randomizer.org). Study participants, study visit clinicians (except for the clinician who prepared the kisspeptin and placebo infusions, but who did not have any contact with the participants or involvement in the data analysis), and hormone analysts were blinded to the identity of the interventions.

All study visits commenced in the morning (ie, at 0800) to control for circadian hormonal changes. Participants were asked to abstain from alcohol and caffeine from midnight the night preceding each study visit, as acute consumption of either substance is known to stimulate the hypothalamic-pituitary-adrenal axis, resulting in cortisol release (43, 44).

Following a brief period of acclimatization, an intravenous cannula was sited in each antecubital fossa for blood sampling and for administration of kisspeptin or placebo. After a further period of acclimatization (to mitigate capturing any confounding effects from cannulation-related stress), heart rate assessment and blood sampling took place at 15-minute intervals from −30 to 75 minutes to measure circulating reproductive hormones and cortisol levels. At time (T) = 0 minutes, a 75-minute intravenous infusion of kisspeptin-54 (1 nmol/kg/h) or placebo (using rate-matched Gelofusine) commenced. The kisspeptin dose was selected to ensure steady-state levels of circulating kisspeptin from 30 to 75 minutes (during the psychometric data collection period), while avoiding downstream sex-steroid increases that would occur later following kisspeptin exposure (25-31). Based on our previous experience, we also know this dose is effective in robustly enhancing sexual and emotional brain processing and resultant behavior in humans (25-31), and so provides a sufficient dose to assess for any potential dynamic effects of kisspeptin on anxiety. Participants completed psychometric questionnaires before and toward the end (ie, T = 70-75 minutes) of the kisspeptin or placebo infusions as detailed later. Blood pressure was measured before and toward the end of the kisspeptin or placebo infusions.

### Outcome Measures

The primary outcome measure was change in behavioral measures of anxiety during kisspeptin compared to placebo administration. The secondary outcome measures were changes in biochemical (as determined by circulating cortisol levels) and physiological (as determined by heart rate and blood pressure assessment) measures of anxiety during kisspeptin compared to placebo administration.

### Kisspeptin-54 Peptide

Kisspeptin-54 was synthesized by Bachem (Bachem Holding AG) and purified by reverse-phase high-performance liquid chromatography. Sterile vials of kisspeptin-54 were produced by Bachem (Clinalfa, Bachem Distribution Services GmbH) according to Good Manufacturing Practice. Electrospray mass spectroscopy and amino acid analysis confirmed the identity of the peptide, as previously described (42). The *Limulus* amebocyte lysate assay (Associates of Cape Cod) was negative for endotoxin, and the peptide was sterile on culture (Department of Microbiology, Hammersmith Hospital, London). Vials of freeze-dried kisspeptin-54 were stored at −20 °C and made up in Gelofusine (B. Braun) and infused at 1 nmol/kg/h, as previously described (25). Placebo (Gelofusine) was administered at a rate equivalent to the kisspeptin infusion and was identical in appearance, thereby respecting the study's double-blinded design.

### Behavioral Assessments

At their first study visit, participants completed a series of well-established and validated psychometric questionnaires to assess relevant baseline traits prior to administration of kisspeptin or placebo (Table 1). The State-Trait Anxiety Inventory (STAI-Y2-Trait) was used to exclude baseline anxiety traits in our cohort (45), with scores of 45 or greater in keeping with clinically high anxiety. The PHQ-9 was used to exclude active depression in our cohort (41), with all scores within normal range (ie, scores 0-4).

**Table 1.** Participant clinical and psychometric characteristics

	Male participants (N = 63)	Female participants (N = 32)	All participants (N = 95)
**Age, y**	31.7 ± 1.2	29.2 ± 1.2	30.9 ± 0.9
BMI, kg/m^**2**^	24.4 ± 0.5	23.1 ± 0.5	24.0 ± 0.4
**Baseline hormone**			
Cortisol, nmol/L*^a^*	351.7 ± 13.7	291.0 ± 25.1	331.3 ± 12.5
LH, IU/L*^b^*	3.2 ± 0.2	6.3 ± 0.6	—
Testosterone, nmol/L*^c^*	19.1 ± 0.7	—	—
Estradiol, pmol/L*^d^*	—	381.1 ± 47.7	—
**PHQ-9** *^e^*	2.3 ± 0.3	2.3 ± 0.4	2.3 ± 0.2
**STAI-Y2-Trait** *^f^*	37.1 ± 1.1	41.0 ± 1.4	38.4 ± 0.9

Data presented as mean ± SEM. N = 95 (N = 63 male participants and N = 32 female participants).

Abbreviations: BMI, body mass index; LH, luteinizing hormone; PHQ-9, Patient Health Questionnaire-9; STAI-Y2-Trait, State-Trait Anxiety Inventory-Y2-Trait.

^a^Serum cortisol. Reference range in nanomoles per liter (nmol/L): 160 to 550.

^b^Serum LH. Reference ranges in international units per liter (IU/L): 2 to 12 (men), 2 to 10 (women, follicular).

^c^Serum testosterone. Reference range in nanomoles per liter (nmol/L): 10 to 30 (men).

^d^Serum estradiol. Reference range in picomoles per liter (pmol/L), 200 to 500 (early follicular).

^e^PHQ-9. Screen for depression (score range, 0-27), with scores 0 to 4 indicating no depression; 5 to 9: mild depression; 10 to 14: moderate depression; 15 to 19: moderately severe depression; and 20 to 27: severe depression.

^f^STAI-Y2-Trait. To assess trait anxiety (score range, 20-80), with scores of 45 or greater indicating clinically significant levels of trait anxiety.

Participants also completed the State-Trait Anxiety Inventory (STAI-Y1-State) before and toward the end of kisspeptin or placebo administration at their first and second study visits (Fig. 1A). This assesses anxiety in the current moment through 20 statements (eg, “I feel tense”), which are answered using a 4-point Likert scale (1 = “not at all,” 2 = “somewhat,” 3 = “moderately so,” 4 = “very much so”) based on how the participant is currently feeling (45). The STAI-Y1-State is regarded as the “gold-standard” (45) and one of the most widely used rating scales for examining acute anxiety symptomatology (46, 47), with robust psychometric properties, including good reliability (48) and validity (49). Importantly, whereas other available anxiety scales such as the Hamilton Anxiety Scale and Beck Anxiety Inventory discriminate poorly between anxiety and depressive symptoms (50-52), the STAI-Y1-State reliably focuses and evaluates the current state of anxiety (45) (ie, the primary objective for the present study). Moreover, unlike other scales, the STAI-Y1-State measures anxiety both in healthy (ie, our intended study population) and in clinical populations (46). A difference of 10% (ie, 8 cumulative points on the STAI) is considered as the minimal clinically important preintervention and postintervention difference for detecting anxiolytic and anxiogenic effects (53). Consistent with this threshold, the STAI-Y1-State has been widely employed in other interventional studies (including Cochrane meta-analyses) and has been shown to detect clinically meaningful anxiolytic effects (eg, in response to β-blockers, benzodiazepines, melatonin, psychedelic drugs, selective serotonin reuptake inhibitors, vitamin D, and nonpharmacological therapies (53-61)), as well as detect clinically meaningful anxiogenic effects (eg, in response to caffeine and cocaine (62, 63)). Therefore, the STAI-Y1-State provided a robust rating scale to assess for any potential dynamic behavioral effects of kisspeptin on anxiety.

### Hormone Assays

Blood samples were collected to measure reproductive hormone and cortisol levels at the time points depicted in Fig. 1A. Plasma kisspeptin immunoreactivity was measured using an established radioimmunoassay with intra-assay and interassay coefficients of variation of 8.3% and 10.2%, respectively, at a limit of detectability of 2 pmol/L (25). Serum LH and testosterone (in men), and estradiol (in women) were measured using automated chemiluminescent immunoassays (Abbott Diagnostics). Serum cortisol was measured using an automated delayed one-step immunoassay (Abbott Diagnostics). Reference ranges: LH in international units per liter (IU/L), 2 to 12 (men), 2 to 10 (women, follicular); testosterone in nanomoles per liter (nmol/L), 10 to 30 (men); estradiol in picomoles per liter (pmol/L), 200 to 500 (early follicular); and cortisol in nmol/L, 160 to 550. Intra-assay and interassay coefficients of variation were as follows: LH, less than 5%; total testosterone, less than 5%; estradiol, less than 5%; and cortisol, less than 10%. Limits of detection for each assay were as follows: LH, 0.07 IU/L; total testosterone, 0.05 nmol/L; estradiol, 70 pmol/L; and cortisol, 22 nmol/L.

### Sample Size

This was a post hoc analysis of 95 male and female participants recruited to research studies examining the effects of kisspeptin administration on human brain processing (25, 30, 31). This is the first dedicated study to investigate the effects of kisspeptin administration on anxiety in men and women. However, previous comparable work examining the acute effects of a hormonal intervention (thyrotropin-releasing hormone) on behavioral measures of anxiety (also determined using the STAI-Y1-State instrument) demonstrated a clinically meaningful reduction in state anxiety scores by mean 7.04 points (SD 2.37), compared to 2.75 points (SD 1.64) following placebo administration (64). Based on these data, a sample size of 16 per group would provide 90% power at an α of .05. Therefore, our sample size of 95 participants should be powered to detect a statistically significant effect of kisspeptin administration on anxiety.

### Statistical Analyses

Statistical analyses were performed using GraphPad Prism (version 10.0). Data are presented as mean ± SEM. Normality of the data was determined using the Shapiro-Wilk test. Time profiles of hormone and heart rate data were analyzed using 2-way analysis of variance with Bonferroni multiple comparison test. As categorical data, differences between baseline and change in psychometric scores during kisspeptin compared with placebo visits were analyzed using Wilcoxon matched-pairs signed-rank test. Difference between baseline and change in blood pressure during kisspeptin compared with placebo visits were analyzed using the paired 2-tailed *t* test. Hormone data are presented as the change from baseline (ie, average of time points −30, −15, and 0 minutes) in circulating levels during kisspeptin, compared with placebo administration. In all cases, *P* less than .05 was considered statistically significant.

## Results

Ninety-five participants (N = 63 male and N = 32 female) completed the study, attending for both a 75-minute intravenous kisspeptin-54 infusion (1 nmol/kg/h) and for a rate-matched placebo infusion (in random order). Participants had a mean ± SEM age of 30.9 ± 0.9 years and a mean body mass index of 24.0 ± 0.4. All participants were free of active anxiety and depression (see Table 1).

### Effects of Kisspeptin Administration on Circulating Reproductive Hormones

**Figure 2.**
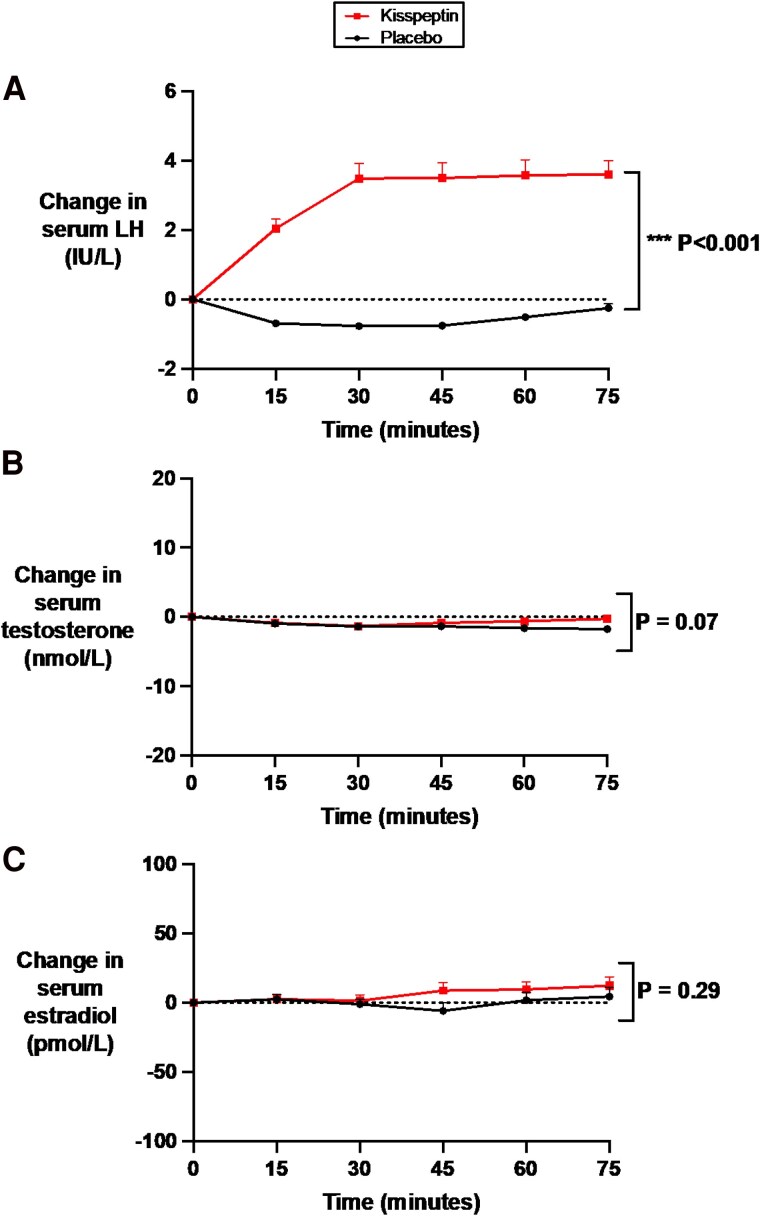
Effects of kisspeptin administration on circulating reproductive hormone levels. Circulating reproductive hormone levels were measured before and at 15-minute intervals during kisspeptin and placebo administration. Kisspeptin administration significantly increased A, circulating luteinizing hormone levels but had no effect on B, downstream testosterone in male participants or C, estradiol in female participants, compared to placebo. Groups were compared by 2-way analysis of variance with Bonferroni multiple comparison test. Data depict mean ± SEM change from baseline (average of time points −30, −15, and 0 minutes). N = 95 (N = 63 male participants and N = 32 female participants).

At baseline (preadministration), kisspeptin, gonadotropin, and downstream sex-steroid levels were equivalent between study visits (Supplementary Table S1) (65). As expected, intravenous kisspeptin administration significantly increased kisspeptin to circulating levels that are known to robustly enhance sexual and emotional brain processing and resultant behavior in humans (25-31), demonstrating that the route and dose of kisspeptin administration was appropriate (see Fig. 1B and Supplementary Fig. S1A and S1B) (65). As a result, kisspeptin administration robustly increased serum LH to similar levels described using this administration protocol (25-31), confirming that the kisspeptin dose used was biologically active (Fig. 2A and Supplementary Figs. S1C and S1D) (65). Kisspeptin administration had no significant effects on downstream sex-steroid levels during the acute 75-minute study period (see Fig. 2B and 2C) in keeping with our previous work (25-31), thereby discounting circulating sex-steroid fluctuations as a possible confounder for any effects on anxiety.

### Effects of Kisspeptin Administration on Behavioral Measures of Anxiety

**Figure 3.**
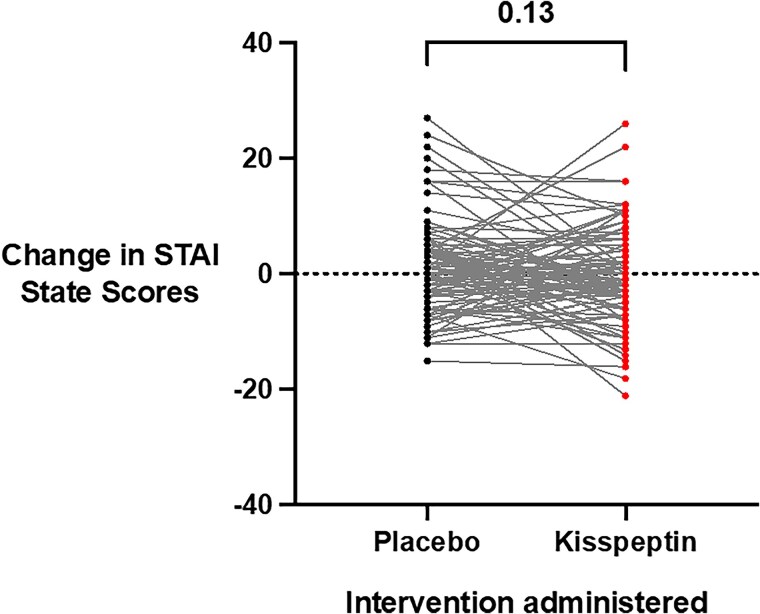
Effects of kisspeptin administration on state anxiety. State anxiety before and during kisspeptin and placebo administration was assessed using the State-Trait Anxiety Inventory (STAI-Y1-State) and was unaltered by kisspeptin administration, compared to placebo (mean difference in STAI-Y1-State scores during infusions: kisspeptin −0.4 ± 0.8 points and placebo 1.3 ± 0.8 points). Total scores ranged from 20 to 80, with higher scores indicating higher levels of anxiety. Presented as score change from baseline for each participant. Groups were compared using Wilcoxon matched-pairs signed-rank test. N = 95 (N = 63 male participants and N = 32 female participants).

Acute (state) anxiety was evaluated using the well-established STAI-Y1-State, which participants completed before and toward the end of the infusions (once steady-state kisspeptin levels had been achieved). Importantly, baseline anxiety scores were equivalent at both study visits before kisspeptin or placebo administration (see Supplementary Table S1) (65). Moreover, there was no statistically significant difference in state anxiety between kisspeptin and placebo (Fig. 3), with kisspeptin's effect comparable both in the male and female participants (Supplementary Fig. S2A and S2B) (65). Taken together, this demonstrates that kisspeptin administration does not acutely affect behavioral measures of anxiety in humans.

### Effects of Kisspeptin Administration on Biochemical Measures of Anxiety

**Figure 4.**
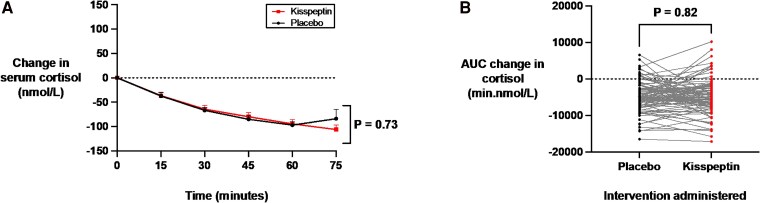
Effects of kisspeptin administration on circulating cortisol levels. A, Circulating cortisol was measured before and at 15-minute intervals during kisspeptin and placebo administration and was unaltered by kisspeptin administration, compared to placebo. Groups were compared by 2-way analysis of variance with Bonferroni multiple comparison test. Data depict mean ± SEM change from baseline (average of time points −30, −15, and 0 minutes). B, Area under the curve (AUC) of the change in cortisol (min.nmol/L) was unaltered by kisspeptin administration, compared to placebo (mean difference in AUC change in cortisol during infusions: kisspeptin −4943.5 ± 500.2 min.nmol/L and placebo −4722.0 ± 440.8 min.nmol/L). Groups were compared by paired *t* tests. N = 95 (N = 63 male participants and N = 32 female participants).

The acute physiological stress response activates the hypothalamic-pituitary-adrenal axis, resulting in elevated circulating cortisol levels. Accordingly, cortisol is a robust biomarker of acute stress and anxiety (66). Therefore, we evaluated the effects of kisspeptin administration on circulating cortisol levels at 15-minute intervals during kisspeptin and placebo administration. Importantly, circulating cortisol levels were equivalent at baseline before kisspeptin or placebo administration (see Supplementary Table S1) (65). We observed that a biologically active dose of kisspeptin did not affect cortisol levels over the 75-minute study period, compared to placebo (Fig. 4A). In addition, further analysis demonstrated no statistically significant difference in the area under the curve for the change in cortisol between kisspeptin and placebo administration (Fig. 4B). Furthermore, the effect of kisspeptin administration on circulating cortisol levels was equivalent in the male and female participants (Supplementary Fig. S3A-S3D) (65). Collectively, this indicates that kisspeptin administration does not have any significant effects on biochemical measures of acute anxiety in humans.

### Effects of Kisspeptin Administration on Physiological Measures of Anxiety

**Figure 5.**
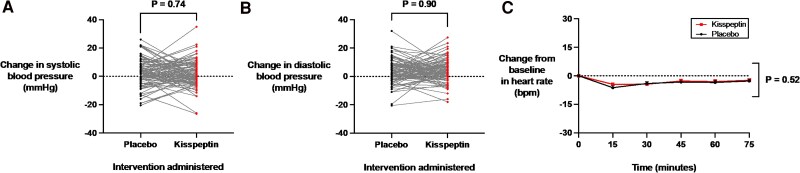
Effects of kisspeptin administration on blood pressure and heart rate measurements. A and B, Blood pressure was assessed before and toward the end of the 75-minute kisspeptin and placebo infusions. Kisspeptin administration had no significant effects on A, systolic (mean difference in systolic blood pressure during infusions: kisspeptin 1.7 ± 0.9 mm Hg and placebo 2.1 ± 1.0 mm Hg) or B, diastolic (mean difference in diastolic blood pressure during infusions: kisspeptin 3.7 ± 0.8 mm Hg and placebo 3.5 ± 0.9 mm Hg) measurements, compared to placebo. Presented as change from baseline for each participant. Groups were compared by paired *t* tests. C, Heart rate was measured before and at 15-minute intervals during kisspeptin and placebo administration and was unaltered by kisspeptin administration, compared to placebo. Groups were compared by 2-way analysis of variance with Bonferroni multiple comparison test. Data depict mean ± SEM change from baseline (average of time points −30, −15, and 0 minutes). N = 95 (N = 63 male participants and N = 32 female participants).

Acute anxiety can manifest with certain well-characterized physiological indicators, including elevations in heart rate and blood pressure (67, 68). We therefore evaluated the effects of kisspeptin administration on heart rate before and at 15-minute intervals during kisspeptin and placebo administration. Blood pressure was also assessed before and toward the end of the 75-minute kisspeptin and placebo infusions. Importantly, heart rate and blood pressure measurements were equivalent at baseline before kisspeptin or placebo administration (see Supplementary Table S1) (65). Here, we observed that kisspeptin administration had no significant effects on systolic (Fig. 5A) and diastolic (Fig. 5B) blood pressure measurements, compared to placebo. Moreover, heart rate was unaltered by kisspeptin administration, compared to placebo (Fig. 5C). Similar to behavioral and biochemical measures of anxiety, kisspeptin's effects on heart rate and blood pressure did not differ significantly between the male and female participants (Supplementary Fig. S4A-S4F) (65). Putting these findings together, this demonstrates that kisspeptin administration had no statisitcally significant clinical effects on heart rate and blood pressure, with increases in these parameters known to be well-established physiological indicators of anxiety in humans.

## Discussion

Here, we report the first study demonstrating that a biologically active dose of kisspeptin administered to men and women does not affect behavioral, biochemical, and physiological measures of anxiety.

Our data provide important translational insights to elucidate kisspeptin's effects on anxiety in humans, particularly given the contradictory preclinical literature. For instance, intracranial administration of kisspeptin to zebrafish (33), as well as selective chemogenetic stimulation of kisspeptin neurons in the amygdala of rodents (23), has been shown to stimulate exploratory behavior and attenuate anxiety. Comparatively, in other rodent studies, central kisspeptin administration was observed not to affect stress-like behaviors, including grooming and locomotive parameters (34), and intraperitoneal kisspeptin shown not to alter basal or stress-induced plasma corticosterone levels (the rodent equivalent of cortisol in humans) (35). In contrast, other research groups have reported that central kisspeptin administration in rodents evoked a preference for the closed arms of the elevated plus maze test and increases corticosterone levels, indicative of anxiogenic effects (36, 38). Given these conflicting findings, our results provide reassurance that acute kisspeptin administration does not affect behavioral, biochemical, or physiological measures of anxiety in humans.

Our demonstration that a single dose of kisspeptin administered to humans does not acutely affect anxiety is of important therapeutic relevance for the development of kisspeptin-based medicines for common reproductive and psychosexual disorders. For instance, we have previously shown that a single dose of kisspeptin induces egg maturation in women with subfertility undergoing in vitro fertilization therapy (11) and stimulates gonadotropin secretion and pulsatility in women with hypothalamic amenorrhea (ie, a common reproductive disorder driven by stress and anxiety) (4, 6), as well as acutely restoring sexual function in patients with low sexual desire, with a proerectile effect in men (30, 31). Moreover, from a diagnostic perspective, a single dose of kisspeptin has diagnostic potential for differentiating common causes of delayed puberty (3) and menstrual disturbance in women (69). In this regard, our data provide reassurance that a biologically active dose of kisspeptin does not acutely affect measures of anxiety. In the context of restoring hormonal secretion in reproductive disorders, chronic (albeit intermittent) administration protocols will be required, whereas for psychosexual disorders an “on-demand’ kisspeptin regimen is more likely. Therefore, although the short investigatory duration employed in the present study precludes us from identifying effects from chronic administration, our data provide reassurance for developing these kisspeptin administration scenarios. Further chronic studies with repeated kisspeptin dosing are warranted to study more chronic effects.

In this study we employed peripheral kisspeptin administration. Data suggest that different kisspeptin isoforms have different degrees of blood-brain barrier penetrance. Peripherally administered kisspeptin-54 (as used in this study) can activate GnRH neuron dendritic terminals outside the blood-brain barrier (2, 70), as well as cross the blood-brain barrier to directly access deeper brain structures implicated in anxiety (such as the hippocampus and frontal lobe) that express kisspeptin receptors (25). Therefore, the route of kisspeptin administration used in this study was sufficient to access brain regions involved in anxiety behavior in humans. Moreover, we administered kisspeptin-54 at a biologically active dose that resulted in circulating kisspeptin levels that are known to be effective in robustly enhancing sexual and emotional brain processing and behavior in humans (25-31). Consistent with this, the evidence of other known biological effects of kisspeptin (ie, increase in LH), allows us to be confident that the route and dose of kisspeptin administration employed was satisfactory to assess for any potential dynamic effects from kisspeptin on anxiety.

This study benefited from several strengths. To comprehensively and robustly investigate the effects of kisspeptin administration on anxiety, we examined well-established and validated behavioral, biochemical, and physiological indicators of anxiety, thereby incorporating similar measures to those used in the earlier discussed preclinical animal studies. Furthermore, we took precautions to reduce sources of variability and control for potential confounders. The crossover design, in which participants acted as their own control, minimized interparticipant variation and enhanced power. Study visits were performed in a randomized order, with participants, study visit clinicians, and data analysts blinded to the intervention administered. Studies commenced in the morning to control for circadian hormonal changes (in reproductive hormones and cortisol), and the kisspeptin administration protocol was selected to avoid downstream sex-steroid increases, thereby excluding these as possible confounders. Regarding the female participants, study visits were matched in terms of the menstrual cycle phase and were all conducted in the follicular phase. This is highly relevant given that fluctuations in reproductive hormones across the menstrual cycle have been shown to be associated with changes in anxiety (71).

Regarding limitations, although this study reports on a large number of participants, the number of male and female participants were not equivalent. Furthermore, it is important clinically to investigate for any sexual dimorphism in response. However, in the subgroup analyses (Supplementary Figs. S2-S4) (65), the behavioral, biochemical, and physiological responses did not differ significantly between male and female participants, suggesting the absence of sexual dimorphism.

In summary, this is the first study demonstrating that a biologically active (pharmacological) dose of kisspeptin to humans does not affect behavioral, biochemical, or physiological measures of anxiety in humans. This provides key clinical data and reassurance that acute kisspeptin administration does not induce anxiety in humans and so informs the rapid development of kisspeptin-based therapeutics for common reproductive and psychosexual disorders.

## Data Availability

Some data sets generated during and/or analyzed during the present study are not publicly available, but are available from the corresponding authors based on reasonable scientific merit. All data provided are anonymized to respect the privacy of the participants.

## Abbreviations

BMI: body mass index
GnRH: gonadotropin-releasing hormone
LH: luteinizing hormone
PHQ-9: Patient Health Questionnaire-9
STAI-Y1-State: State-Trait Anxiety Inventory-Y1-State
STAI-Y2-Trait: State-Trait Anxiety Inventory-Y2-Trait
